# Pelvic floor function after third and fourth degree perineal lacerations: a case-control study on quality of life

**DOI:** 10.1186/s12905-023-02739-9

**Published:** 2024-01-03

**Authors:** Andrea Sartore, Maria Sole Scalia, Francesco Paolo Mangino, Giulia Savastano, Elena Magni, Giuseppe Ricci

**Affiliations:** 1 Institute for Maternal and Child Health-IRCCS “Burlo Garofolo”, 34137 Trieste, Italy; 2 Department of Medicine, Surgery and Health Sciences, University of Trieste, 34127 Trieste, Italy; 3 Clinical Epidemiology and Public Health Research Unit, Institute for Maternal and Child Health-IRCCS “Burlo Garofolo”, Trieste, Italy

**Keywords:** Pelvic floor dysfunction, Quality of life, OASI, Perineal tears

## Abstract

**Background:**

The primary aim of this study was to compare the quality of life between women with obstetric anal sphincter injury (OASI) and women with intact perineum or minor vaginal tears following their first vaginal birth through a validated urogynaecological questionnaire. As a secondary aim, we wanted to identify the specific symptoms for pelvic floor dysfunction after a vaginal birth.

**Methods:**

One hundred thirty-three cases (III- and IV-degree vaginal tears) and 133 controls (intact perineum or I- and II-degree vaginal tear) were asked to fill the PFDI-20 condition-specific and quality of life survey at three and 12 months after vaginal delivery. The survey evaluates pelvic floor dysfunction symptoms through three subsections: the Pelvic Organ Prolapse Distress Inventory (POPDI), the Colorectal-Anal Distress Inventory (CRADI), and Urinary Distress Inventory, (UDI). The scoring system ranges from 0 (no distress) to 100 (maximum distress) for each subsection, subsequently summed up to obtain the summary score (0 to 300). The patients recruited were asked to complete the survey at 3- and 12-months follow-up visit. Accordingly, data collection started. Categorical variables were subjected to Chi-square test or Fisher’s Exact test. Quantitative variables were compared through Student’s t-test or Mann-Whitney test.

**Results:**

All surveys have shown statistically significant differences when comparing the cases to the control group. Consequently, PFDI-20 has shown a strong correlation between III- and IV-grade lacerations and pelvic floor dysfunction persistence at 12 months after delivery. Intestinal symptoms were the most reported disturbances among women with previous OASI.

**Conclusions:**

Major vaginal tears have demonstrated to have a strong impact on women’s quality of life up to a follow-up of 12 months. The use of PFDI-20 questionnaire is a useful and valid tool in the diagnosis and follow-up of genital prolapse, fecal and urinary incontinence in primiparous women with a history of OASI. Thus, its application in clinical practice can help offering the most adequate rehabilitative treatment.

## Background

It is estimated that 85% of women who deliver spontaneously can face any form of vaginal tear, usually with incidences decreasing in subsequent births [1].

Anterior perineal trauma can involve the vaginal wall, urethra, clitoris, and labia. Posterior perineal trauma is more frequent and can affect the posterior vaginal wall, perineal muscle and body, the external and internal anal sphincters, up to the anorectal mucosa [2]. The injuries can result in disabling immediate complications such as hemorrhage, infection, wound dehiscence, and long-term complications represented by pelvic floor prolapse, urinary and fecal incontinence, dyspareunia, and chronic pelvic pain [3].

In 1999 Sultan [4] has formulated the following classification, currently adopted both by the International Consultation on Incontinence and the Royal College of Obstetricians & Gynaecologist (RCOG). In summary, first degree tear: injury to perineal skin and/or vaginal mucosa; second-degree tear: injury to perineum involving perineal muscles but not involving the anal sphincter; third degree tear: injury to perineum involving the anal sphincter complex (IIIA: less than 50% of external anal sphincter – EAS - thickness torn; IIIB: more than 50% of EAS thickness torn; IIIC: both EAS and internal anal sphincter – IAS - torn); fourth degree tear: injury to perineum involving the anal sphincter complex (EAS and IAS) and anorectal mucosa. Third- and fourth-degree perineal tears have an incidence in primiparous women of 3–6.5% [5–8] and are a frequent cause of pelvic floor dysfunction, fecal and urinary incontinence, dyspareunia, and pain with symptoms that can persist for many years after vaginal delivery. Incidence rates vary widely between countries [9], and in England incidence has tripled from 1.8% in 2000 to 5.9% in 2012 [10]. In Italy, the College of Health (https://www.epicentro.iss.it/territorio/trento/pdf/Report_Natalita_2018.pdf.) has registered a rate of perineal tears from vaginal births rising between 2000 and 2018, with a peak of 72% in 2018.

Risk factors are multiple and object of discussion in different studies. Maternal risk factors that can be considered are nulliparity [11], advanced maternal age (> 35 years) due to physiologic connective tissue changes [12], and vulvar-anal length below 4 cm [13]. Among fetal risk factors we recall macrosomia (seen as fetal weight > 4 Kg) [14], occipito-posterior position, and shoulder dystocia [15]. Known iatrogenic risk factors are labor induction and augmentation, instrumental delivery, and prolonged second stage of labor [16, 17].

Between 60 to 80% of women are asymptomatic at 1 year follow up, if an early recognition of major perineal tears and adequate surgical repair through overlap or end-to end technique are made [10, 18]. However, despite improvements in prevention and treatment techniques in recent years, many women still suffer from a negative impact on life quality after giving vaginal birth. The quality of life is an aspect difficult to interpret and with multiple connotations, frequently underestimated and complex to study. Validated questionnaires can categorize the different social areas of major impact on women lives by recognizing specific pathology-related symptoms. With this study we want to investigate the difference in term of quality of life between women with an OASI and women with intact perineum or minor vaginal tears (I or II grade) following their first vaginal delivery. Specifically, we want to identify the features of pelvic floor dysfunction according to the grade of vaginal tear through a validated pathology-related questionnaire that can be a support in patients’ treatment and follow-up.

## Methods

This is a prospective qualitative case-control study carried out on 266 spontaneous vaginal deliveries from 2015 to 2021 in the Department of Obstetrics and Gynaecology of the University Hospital “IRCCS Burlo Garofolo” of Trieste, Italy. The recruitment of the patients started after delivery at discharge from the Hospital. Inclusion criteria were age 18 or older, first pregnancy with vaginal delivery and major perineal tear (grade III or IV) for cases; intact perineum or minor vaginal tear (grade I or II) for controls. Perineal tears were all classified according to Sultan [4]. All minor tears included in the study didn’t involve levator ani injury. Exclusion criteria were: more than one previous vaginal delivery and/or pregnancy, instrumental delivery (vaginal vacuum or forceps), episiotomy, pre-existing pelvic floor dysfunctions. All patients recruited underwent a follow-up visit at three and 12 months. Before starting the visit, patients were asked to fulfill the validated questionnaire Pelvic Floor Disability Index (PFDI-20). For each patient recruited with a major vaginal tear, we recruited a successive patient with a minor vaginal tear or intact perineum.

Baseline characteristics analyzed for both cases and controls were: age, preconception body mass index (BMI), excessive weight gain in pregnancy, gestational diabetes mellitus (GDM), baby birth weight, smoking. According to the ACOG (American College of Obstetricians and Gynecologists) Guidelines [19], excessive weight gain in pregnancy was assessed as follows: for patients underweight (BMI ≤ 18.5) weight gain (WG) was excessive when exceeding 18 Kg, for those normal-weight (BMI 18.5–24.9) when WG > 16 Kg, for those overweight (BMI 25–29.9) when WG > 11 Kg, for those obese (BMI > 30) when WG > 9 Kg.

We firstly recruited 398 patients: 199 in the case and 199 in the control group. We encountered 66 dropouts in the case and 48 in the control group, for a total of 114 dropouts. Of these, 17 and 74 patients didn’t show up at respectively three- and 12-months follow-up visit. Moreover, 23 patients refused reporting the answers because they felt uncomfortable referring such sensitive topics. To keep the population comparable between the two groups, we randomly excluded 18 controls. Therefore, we recruited 133 patients in the case group and 133 patients in the control group.

The PFDI-20 survey is a validated [20] condition-specific and life quality questionnaire that evaluates symptoms related to pelvic floor dysfunction. It consists of 20 questions divided in three parts: the first one studies symptoms specific for pelvic prolapse (Pelvic Organ Prolapse Distress Inventory, POPDI, involving 6 questions), the second one investigates intestinal symptoms related to pelvic prolapse (Colorectal-Anal Distress Inventory, CRADI, involving 8 questions), and the third one evaluates urinary symptoms related to pelvic prolapse (Urinary Distress Inventory, UDI, involving 6 questions). Each question takes up an answer that indicates the severity of the symptom on a scale from 0 to 4: “0” (no symptom), “1” (occasional disturbance), “2” (disturbance present but does not affect daily activities), “3” (disturbance almost continuous which sometimes influences daily activities), “4” (persistent disturbance which influences daily activities).

The aim of our study was to evaluate the quality of life of women with major perineal tears as compared to those with intact perineum or with minor perineal damage using a validated and pathology-specific questionnaire. As a secondary aim, we wanted to identify the specific symptoms for pelvic floor dysfunction after a vaginal birth.

The study was previously approved by our local ethical committee.

## Statistical analysis

A summary score was computed for each of the three sections of the PFDI-20 multiplying individuals’ average scores by 25 as to obtain values ranging from 0 (minimal distress) to 100 (greatest distress). The sum of these scores defines the overall summary score, which ranges from 0 to 300 [20]. Data were reported as number and percentage for categorical variables and Chi-square test or Fisher’s Exact test, when appropriate, were used to compare the group of cases with the group of controls. Quantitative variables were expressed as mean and standard deviation for normally distributed variables or as median and interquartile range otherwise and the independence between the group of cases and the group of control was assessed using Student’s t-test or Mann-Whitney test, respectively. Statistical significance was considered with a p- value set below 0.05. All statistical analysis was performed using R Software, Version 4.1.1 (R Foundation for Statistical Computing, Vienna).

## Results

**Fig. 1:**
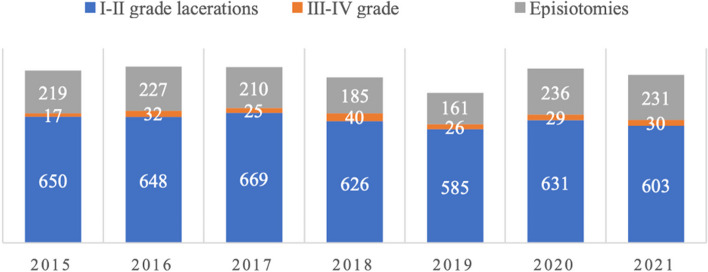
Time-trend distribution of I-II grade, III-IV grade vaginal tears and of episiotomies (years 2015–2021)

In the years 2015–2021 we registered at our Department a total of 10,361 deliveries: 7416 (71.6%) spontaneous, 911 (8.8%) instrumental, and 2034 (19.6%) cesarean sections. The number of III and IV grade tears was 199 (2.4% of all vaginal births). The number of episiotomies was 1469 (17.6% of all vaginal births). The trend of distribution of perineal tears and episiotomies in the years considered is resumed in Fig. 1.

Baselines population characteristics are resumed in Table 1. Average age at delivery of patients included in the study was 32.8 ± 5.1 years Among cases, patients’ preconception BMI was averagely higher than controls (*p* < 0.001). Similarly, cases showed more weight gain in pregnancy (*p* = 0.002), more GDM (NS), and higher baby birth weight (*p* < 0.001) as compared to controls. Moreover, smokers were slight more prevalent among cases rather than controls (NS).

**Table 1:**
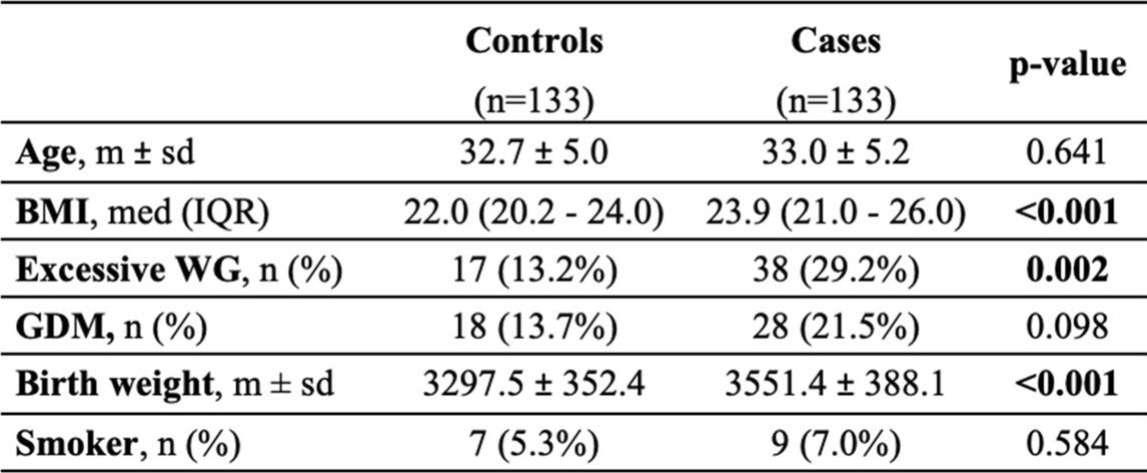
Baseline characteristics

BMI=Body Mass Index; IQR: interquartile range; WG: weight gain; GDM: gestational diabetes mellitus

The distribution of the lacerations between the groups is illustrated in Table 2.

**Table 2:**
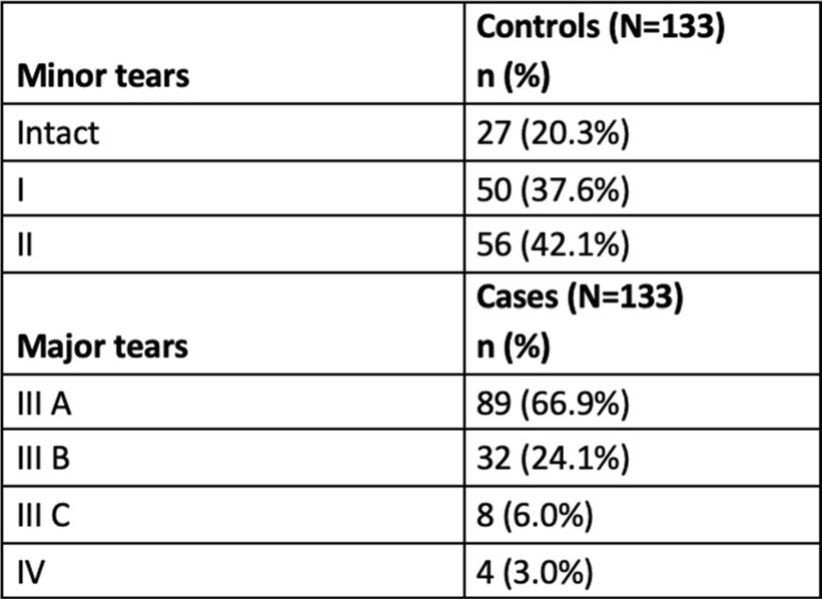
Type of tear in the control and in the case group

The analysis of the results for POPDI-6 at 3 months follow-up has shown that cases are significantly more symptomatic when compared to the control group as they were complaining about pressure in lower abdomen and heaviness or dullness in the pelvic area. Moreover, cases were more symptomatic at 3 months follow-up as complaining about feeling of incomplete bladder emptying (Table 3).

**Table 3:**
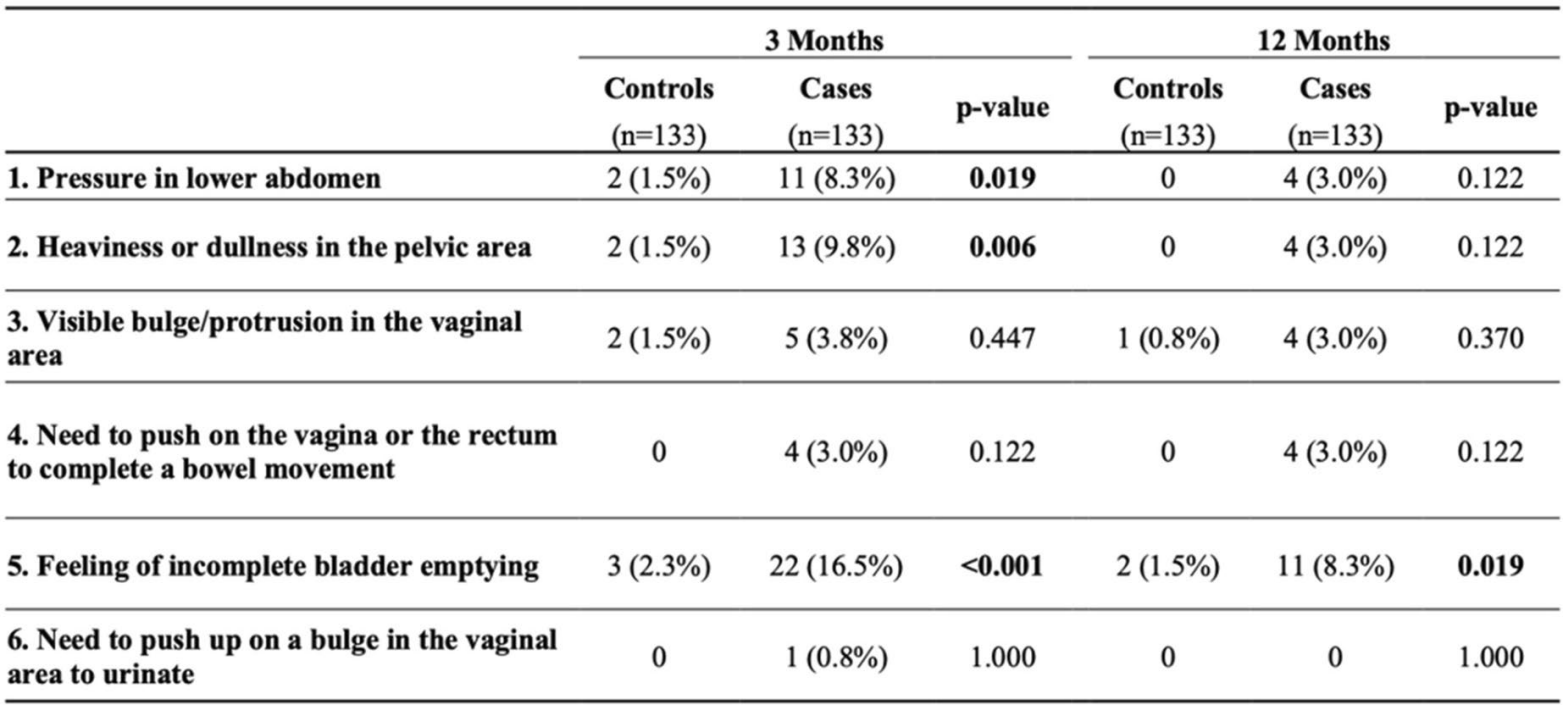
Proportion of individuals who scored 3–4 for each item of the POPDI-6 survey

The results of CRAD-8 have shown that cases were more symptomatic than controls at 3 months follow-up as they reported need to strain too hard to have bowel movements, feeling of incomplete bowel emptying, losing of loose stool beyond control, losing gas from rectum beyond control, pain with stool passing, and strong sense of urgency to have a bowel movement. At 3 months follow-up they kept reporting feeling of incomplete bowel emptying, losing gas from rectum beyond control and strong sense of urgency to have a bowel movement (Table 4).

**Table 4:**
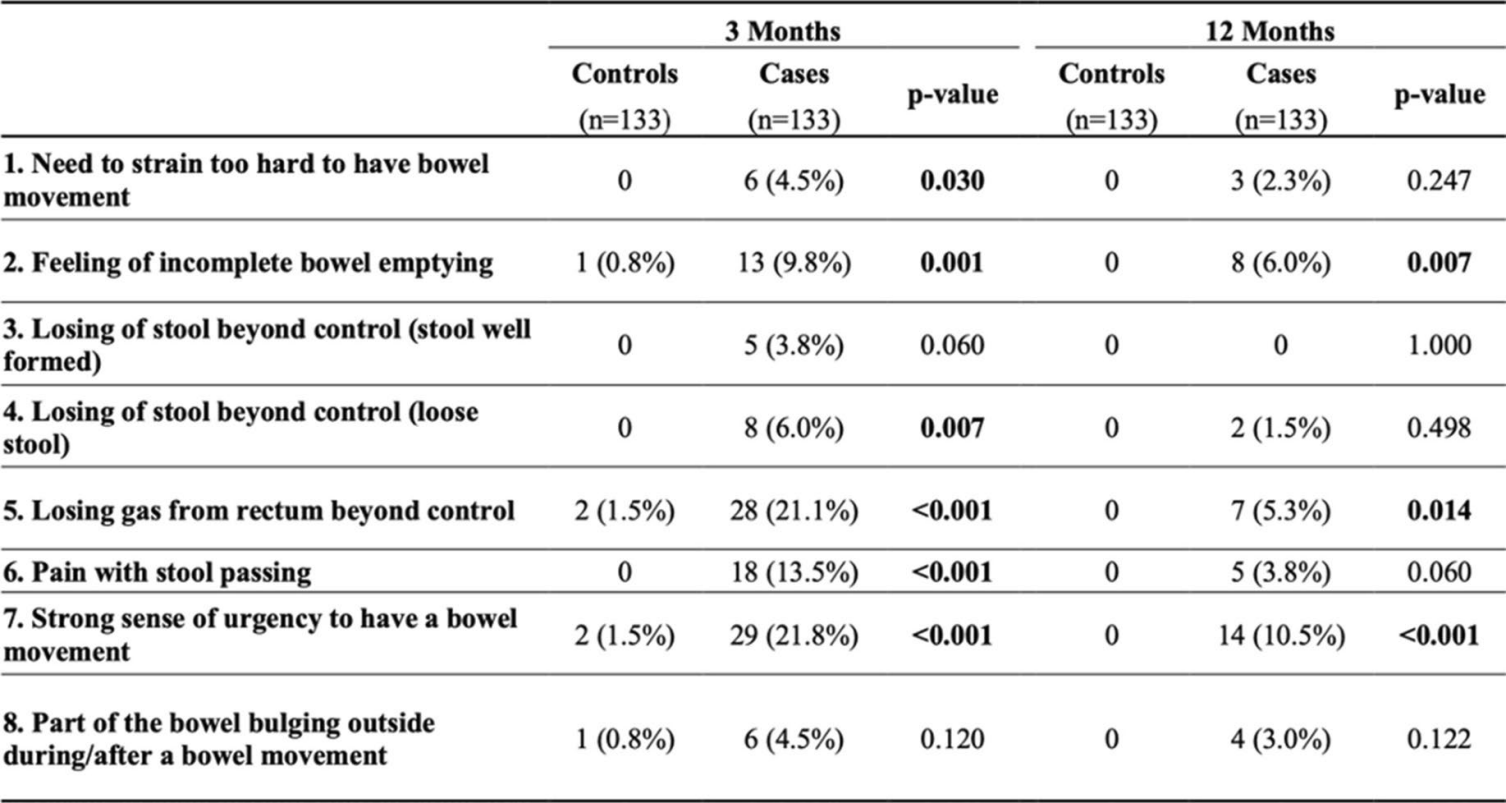
Proportion of individuals who scored 3–4 for each item of the CRADI-8 survey

The questionnaire UDI-6 has shown statistical significance among cases at 3 months follow-up as they reported frequent urination, urine leakage associated with a feeling of urgency and urine leakage related to coughing, sneezing, or laughing. At 12 months they kept reporting frequent urination and urine leakage related to coughing, sneezing or laughing (Table 5).

**Table 5:**
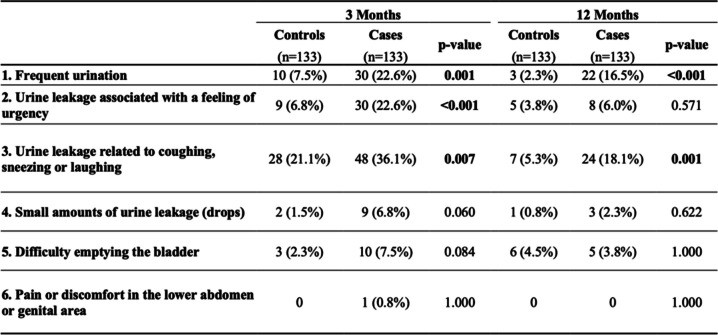
Proportion of individuals who scored 3–4 for each item of the UDI-6 survey

**Fig. 2:**
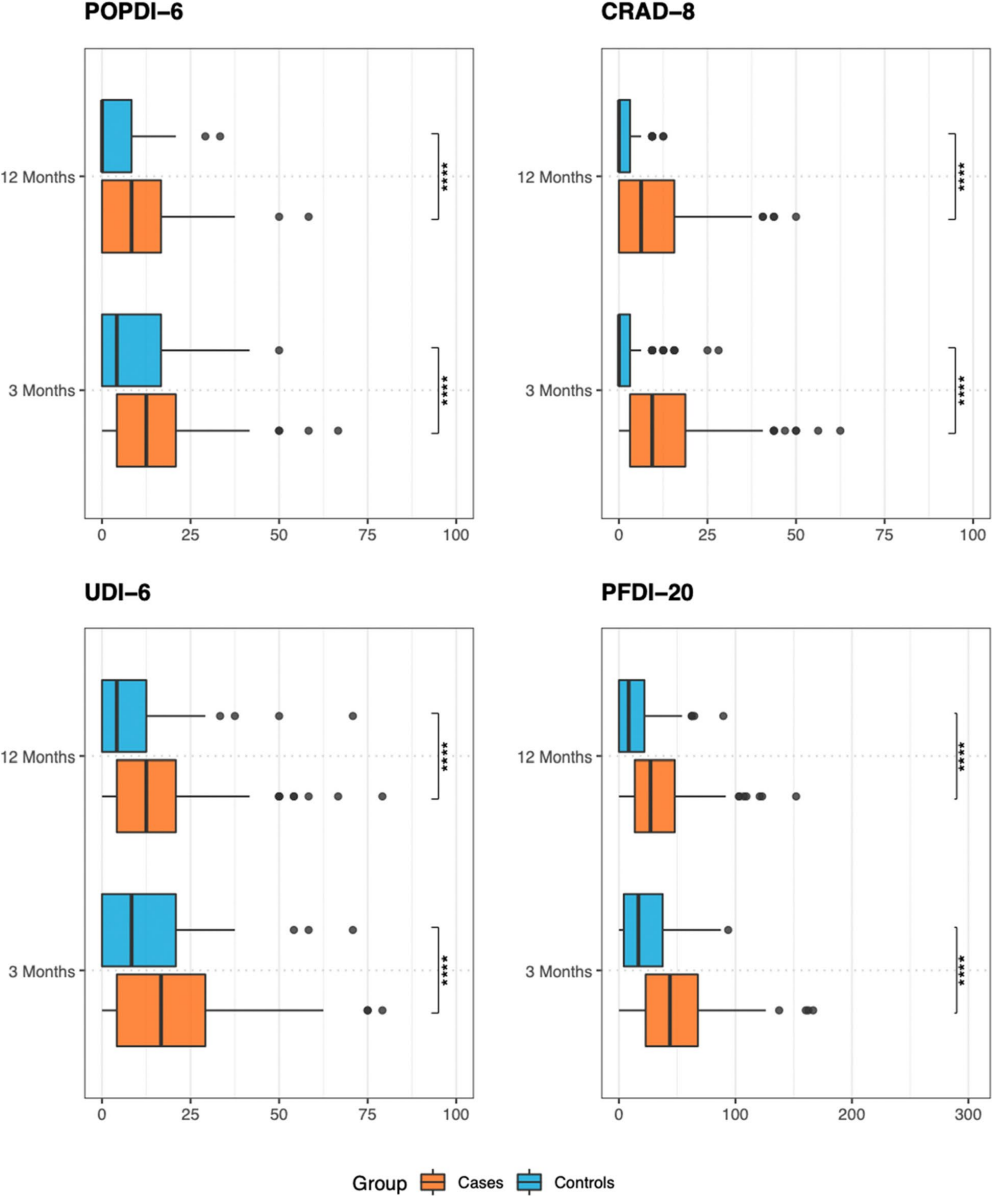
Comparison at 3- and 12-months follow-up between cases and controls with Student’s t-test significance level. The four stars (****) stand for a *p* ≤ 0.0001

In Fig. 2 we show the comparison between cases and controls for the Summary Score of each survey at three and at 12 months follow-up. All questionnaires have shown to be statistically significant when cases were compared to controls at both three- and 12-months follow-up (Table 6).

**Table 6:**
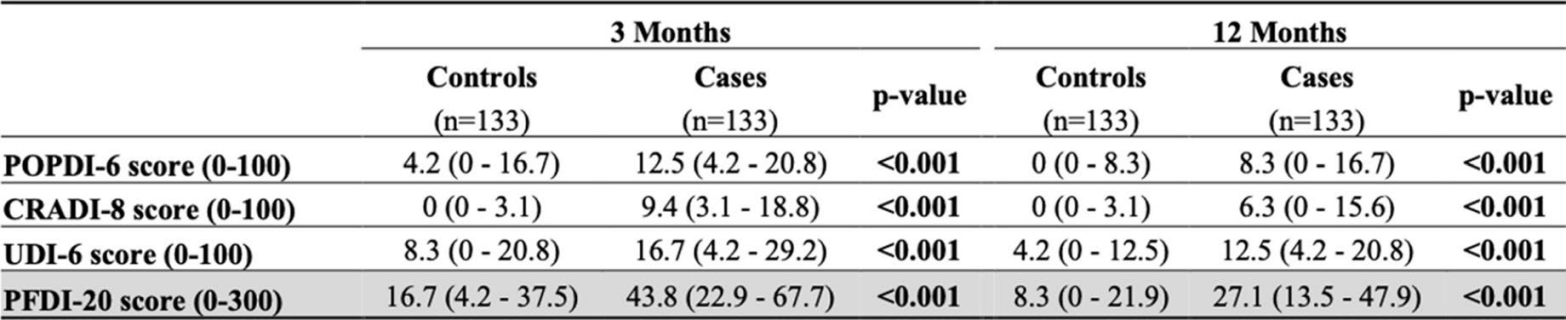
Summary scores written ad median and interquartile range

## Discussion

The present study demonstrates that PFDI-20 is a valid and useful tool for the analysis of pelvic floor dysfunction among the puerperal population through quality-of-life assessment. Perineal tears are a common event after a vaginal birth with incidences ranging between 53 and 85%, widely depending on patients’ characteristics, delivery dynamics and obstetric assistance given during labor [13]. According to our case study, major vaginal injuries were indeed more prevalent among women with high pre-gestational BMI, excessive weight gain during pregnancy and higher fetal birth weight.

Most of vaginal tears are minor (I and II grade) and heal without consequences [16]. However, injuries involving the anal sphincter affect from 3 to 11% of nullipara women after vaginal birth [5–10] and are associated with significant short and long term maternal morbidity. Similarly, in our case history the rate of perineal tears of births registered between 2015 and 2021 was of 2.4% for III and IV degree and of 53% for I and II degree.

Although most of the women (60–80%) are asymptomatic at 1 year follow-up [10], the damage at the complex of the anal sphincter can still be the cause for pelvic floor dysfunction, such as pelvic floor prolapse and urinary incontinence, with a grade of severity that is scarcely commented in the different studies. In fact, as shown in a recent study involving 1348 women over a follow up of 13 years through anorectal noninvasive testing of asymptomatic women following primary OASI, occult damage is not infrequent [21] and, above all, fecal incontinence can go unnoticed for several years before getting to the physician’s attention, eventually leading to a poor quality of life many years later [22].

Levator ani avulsion is the result of an excessive mechanical stretch and distension during delivery. The damage could be mild or severe, frequently depending on the laterality of the injury (unilateral and bilateral avulsion, respectively) [23]. As a consequence of the muscular avulsion, the gap between the levator ani insertion on the inferior pubic ramus and the centre of the urethra lumen has been demonstrated to be widened [24]. In fact, the levator-urethra-gap (LUG) has from different years been proposed as a landmark for a qualitative and objective evaluation of the local injury, either through a clinical (vaginal palpation) or radiological evaluation (translabial ultrasound reconstruction and magnetic resonance imaging, respectively) [24–26].

Urinary and fecal incontinence are the cause of important embarrassment and social discomfort in a woman’s life and, as previously stated, are difficult to investigate during post-partum control visits. Hence the need of relying on validated questionnaires among the puerperal population that permit to categorize and subsequently follow over time the course of pathology specific symptoms.

The survey PFDI-20 was introduced in 2001 to measure the quality of life in women with pelvic floor disorders to evaluate the efficacy of a specific therapy or to compare symptoms among different groups of patients. The questionnaire followed further surveys, firstly described by Shumaker et al. [27], made for women with lower urinary tract dysfunction (UDI-Urinary Stress Inventory and IIQ-Incontinence Impact Questionnaire) [20].

Although these tools have been generally applied on symptomatic populations, our study is the first that offers PFDI-20 to a group of women who have recently given birth, regardless of their symptomatology. In fact, perineal examination alone hasn’t demonstrated to be a good predictor for the whole spectrum of symptoms related to pelvic floor dysfunction [28]. For these reasons, we believe PFDI-20 provides additional information to identify and stratify possible disorders according to a certain degree of vaginal tear.

Despite the direct relationship between perineal damage severity and grade of reported discomfort has been subject of debate [29–31], our study has shown that most of pelvic floor disturbances tend to be worsening or persisting during time in the group of severe perineal tears rather than in the control group.

According to K.L. Shek [31], one of the hypotheses in support of genital prolapse onset is that III- and IV- grade vaginal tears are more frequently associated to occult avulsion of elevator ani muscle. In fact, our study confirms that women with major perineal damages experience more frequently symptoms of organ prolapse such as pressure, heaviness, and dullness in the lower abdomen.

On the other hand, intestinal symptoms related to pelvic prolapse have shown to have an import impact on daily life of women with previous major lacerations. These patients have demonstrated to suffer from lack of defecatory stimulus, incomplete intestinal voiding, gas and fecal incontinence, pain and defecatory urgency, meteorism and genital bulging burden.

These results agree with a systematic review [32] showing that III- and IV-degree sphincter rupture is the only etiological factor strongly (complete anal incontinence) or moderately (flatus incontinence) associated to postpartum fecal incontinence. Therefore, from our point of view the CRADI subsection is a fundamental tool in evaluating and following during time patients with vaginal-sphincter tears.

A cross-sectional study conducted 3 months after delivery on 537 women, showed that the prevalence of urinary symptoms in primiparas was of 8.2% and of 5.5% for, respectively, stress and urge incontinence, resulting in a significant correlation between urinary frequency and dysuria and increased number of subsequent vaginal births [33].

In our study, among urinary symptoms severe perineal tears were associated more frequently to pollakiuria, urge and stress incontinence, and incomplete vesical emptying. Effectively, it is estimated that during the first 3 months postpartum, the prevalence of any postpartum incontinence is as high as 33% and longitudinal studies within the first year postpartum have reported only small changes of prevalence over time [32]. Maternal age and nulliparity are the factors with major impact on this aspect [34].

Therefore, our study confirms what already known about the tight relationship between vaginal delivery, severe perineal tear, and pelvic floor dysfunction. The statistical significance of most of the items analyzed for each survey seems to support the importance of PDFI-20 as a tool in the diagnosis and the follow-up of pelvic floor disorders in nulliparae women with a recent first vaginal birth complicated with III- or IV-degree perineal tear.

## Strenghts and limitations

The strength of the study is its novelty in introducing a validated quality of life questionnaire in a selected puerperal population. A limitation of the study is the short follow-up period of the patients included in the investigation.

## Conclusions

Vaginal delivery can be the cause of severe damage on pelvic floor tissue. Perineal III- and IV- grade vaginal tears have an important impact on life quality of women in the first 12 months postpartum period. In these patients the use of the validated PFDI-20 survey has demonstrated to be a valid and helpful tool in the diagnosis and follow-up of genital prolapse, fecal and urinary incontinence. By helping investigate a wide range of symptoms that can frequently remain under-dimensioned, the survey could be used in clinical practice to tailor the best rehabilitative treatment to each patient.

## Data Availability

The authors confirm that the data supporting the findings of this study are available within the article.

## Abbreviations

OASI: obstetric anal sphincter injury
EAS: external anal sphincter
IAS: internal anal sphincter
PFDI: Pelvic Floor Disability Index
POPDI: Pelvic Organ Prolapse Distress Inventory
CRADI: Colorectal-Anal Distress Inventory
UDI: Urinary Distress Inventory
